# The association between benzodiazepine and non-benzodiazepine and suicide: a nationwide cohort study

**DOI:** 10.1192/j.eurpsy.2022.478

**Published:** 2022-09-01

**Authors:** N. Høier, T. Madsen, A. Spira, K. Hawton, P. Jennum, M. Nordentoft, A. Erlangsen

**Affiliations:** 1 Mental Health Centre Copenhagen (CORE), Danish Research Institute For Suicide Prevention, Hellerup, Denmark; 2 Mental Health Center Copenhagen, Core-copenhagen Research Center For Mental Health, Hellerup, Denmark; 3 Johns Hopkins Bloomberg School of Public Health, Department Of Mental Health, Baltimore, United States of America; 4 University of Oxford, Centre For Suicide Research, Oxford, United Kingdom; 5 Danish Center for Sleep Medicine, Rigshospitalet, Copenhagen University, Copenhagen N, Denmark; 6 CORE-Copenhagen Research Center for Mental Health, Mental Health Center Copenhagen, Hellerup, Denmark

**Keywords:** Suicide, sleep medicine, Pharmacology, benzodiazepine

## Abstract

**Introduction:**

Benzodiazepines and non-benzodiazepines have been linked to a variety of adverse effects including addiction. Long term use of these drugs has been associated with an increased risk of suicide.

**Objectives:**

We assessed if individuals in treatment with non-benzodiazepine (n-BZD) and benzodiazepine (BZD) had higher rates of suicide when compared to individuals not in treatment with these drugs.

**Methods:**

We utilized a cohort design and national longitudinal data on all individuals aged 10 or above who lived in Denmark between 1995 and 2018. Treatment with either n-BZD or BZD was identified via the Danish National Prescription Registry and suicide deaths were identified in the national cause of death registries.

**Results:**

In a total of 6,494,206 individuals, 10,862 males and 4,214 females died by suicide. Of these, 1,220 (11.2%) males and 792 (18.8%) females had been in treatment with n-BZD, resulting in adjusted IRR for suicide of 4.2 (95% CI, 4.0 – 4.5) and 3.4 (95% CI, 3.1 – 3.7) for males and females, respectively, when compared to those not in treatment. In all, 529 (4.8%) males and 395 (9.3%) females who died by suicide had been in treatment with BZD. The IRRs for suicide were 2.4 (95% CI, 2.2 – 2.6) and 2.5 (95% CI, 2.3 – 2.8) for males and females, respectively, and compared to those not in treatment.

**Conclusions:**

In this study we find that those in treatment experienced higher suicide rates than those not in treatment, this persisted when also adjusting for a large variety of covariates.

**Disclosure:**

No significant relationships.

